# Scaling up long-acting reversible contraception through task sharing and capacity building: an implementation science approach in Balsas, Brazil

**DOI:** 10.3389/fgwh.2026.1673405

**Published:** 2026-04-13

**Authors:** Moazzam Ali, Adriano Bueno Tavares, Liana Bastos Matos Modesto, James Kiarie

**Affiliations:** 1 UNDP/UNFPA/UNICEF/WHO/World Bank Special Programme of Research, Development and Research Training in Human Reproduction (HRP), Department of Sexual and Reproductive Health and Research, World Health Organization, Geneva, Switzerland; 2 Family, Health Promotion and Life Course, World Health Organization, Mexico City, Mexico; 3 Dr. Rosy Kury Municipal Hospital, Balsas, Brazil

**Keywords:** capacity building, contraception, implementation science, IUD, task sharing, Brazil

## Abstract

**Introduction:**

The 2015 Zika outbreak crises in Brazil, exposed major challenges in access to contraception services. This report examines outcomes of project aimed at strengthening and studying family planning services in Balsas, Maranhão, an area severely affected by ZIKV.

**Methods:**

The project was guided by the Exploration, Preparation, Implementation, Sustainment (EPIS) framework. It followed a structured, four-phase process to systematically design, implement, and sustain evidence-based interventions aimed at enhancing contraceptive service delivery. Key activities included stakeholder engagement, health system assessments, capacity building, cascade training and task sharing from specialists to general practitioners and nurses.

**Results:**

The intervention led to significant improvements in utilization of family planning services. The transformation of the Women's Health Program, based at the Dr. Rosy Kury Municipal Hospital in Balsas, into a center of excellence, enabled the introduction and scale-up of previously unavailable services such as IUD insertion and removal. This resulted in approximately 1,468 voluntary IUD insertions within 1 year, a dramatic increase from only eight in the previous 5 years. Task sharing IUD procedures with general practitioners and nurses significantly expanded service coverage. Comprehensive training and mentoring were extended to all 26 primary care facilities in Balsas and to providers in four neighboring municipalities, with a total of 80 providers trained, contributing to increased contraceptive uptake across the region.

**Conclusion:**

The structured implementation approach effectively addressed systemic barriers to contraceptive access in a resource-limited setting. By empowering general practitioners and nurses to deliver a broader range of contraceptive methods, the intervention significantly enhanced service delivery. The success of this model highlights its potential for replication in similar contexts and underscores the importance of ongoing capacity building and strengthened health information systems to sustain long-term improvements.

## 1 Introduction

The Zika virus (ZIKV) is a mosquito-borne pathogen of international public health importance due to its teratogenic potential (1). ZIKV infection during pregnancy can result in severe fetal outcomes including microcephaly, brain anomalies, ocular defects, and pregnancy loss (2). First reported in Brazil in May 2015, the Zika outbreak affected an estimated 440,000 to 1.3 million individuals in Brazil and other countries (3).

Zika's impact underscored critical gaps in access to reproductive health services, especially contraception. The epidemic highlighted the need for effective family planning, as several governments, including Brazil's, advised women to delay or avoid pregnancy. However, this advice was not supported by an adequate expansion in contraceptive service availability, particularly in underserved areas.

Brazil, a country of approximately 214 million people, has a modern contraceptive prevalence rate (mCPR) of 63% among all women, with an unmet need for contraception estimated at 6% (4, 5). Approximately 89% of total demand for contraception is met by modern methods (6), yet an estimated 50%–55% of all births in the country are unplanned, underscoring persistent barriers to access and quality in family planning services (7–10). While Brazil's national unmet need for contraception is relatively low (6%), the high proportion of unplanned pregnancies suggests significant gaps in access to effective methods, inconsistent use, and misalignment between contraceptive methods and user preferences. This project specifically targeted the underutilization of long-acting reversible contraceptives, particularly IUDs, and the limited availability of IUD services outside specialized urban centers. These unintended pregnancies impose substantial financial and social costs on families as well as on the public healthcare system (11).

The 1988 Brazilian Constitution recognized health as a universal right, leading to the establishment of the Sistema Único de Saúde (SUS), a publicly funded and universally accessible health system that now serves approximately 75% of the population (12, 13). Although contraception is offered free of charge through SUS, access to the full range of methods remains uneven, especially in underserved and rural regions (14, 15). Currently, nine contraceptive methods are routinely available through SUS: combined and progestin-only oral contraceptives, injectables, male and female condoms, diaphragms, emergency contraception, and copper intrauterine devices (IUDs) (16). Despite this availability, long-acting reversible contraceptives (LARCs), including hormonal implants and IUDs remain significantly underutilized. For instance, copper IUD use stands at just 1.8% among women of reproductive age (17). Hormonal implants are limited to the private sector, and female sterilization is largely confined to urban tertiary-level healthcare facilities, further limiting equitable access to long-term contraceptive options (18).

Several barriers hinder LARC uptake, including provider training gaps, limited service availability outside urban centers, and widespread misconceptions among potential users. In Brazil, copper IUDs can only be inserted or removed by trained gynecologists, further restricting access in lower-level facilities (19, 20).

Despite these challenges, family planning remains a cost-effective health intervention. Recognizing the opportunity to address persistent gaps in access to long-acting reversible contraception -particularly the severe underutilization of IUDs (1.8%) despite high levels of unplanned pregnancy—the Ministry of Health, in collaboration with WHO, launched a targeted initiative in Balsas, Maranhão. The project aimed to strengthen health system capacity for LARC provision, implement task sharing to expand service coverage, and improve quality of contraceptive counseling to support informed method choice (21, 22).

## 2 Methods and study site

In the context of Zika's emergence and its implications for reproductive health, Brazil's Ministry of Health, in partnership with WHO, initiated a project to strengthen and study family planning services in underserved areas. Balsas, located in the state of Maranhão, was selected due to its high ZIKV incidence and limited capacity to provide contraceptive services. The project aimed to identify health system bottlenecks and expand access to modern contraceptive methods using an implementation science framework.

The intervention was implemented from June 2017 to December 2018, spanning approximately 18 months. Training activities began in August 2017, with cascade training continuing through December 2018.

As of 2021, located in northeastern Brazil, Maranhão continues to be Brazil's most socioeconomically vulnerable state, with a municipal Human Development Index of 0.676—the lowest of all 27 states. Maternal mortality remains a critical challenge: in 2020, the state recorded an MMR of 109 deaths per 100,000 live births, among the highest in the Northeast. In 2021, impacted substantially by the COVID-19 pandemic, Brazil's national MMR surged to over 113 per 100,000, with the Northeast continuing to bear one of the region's highest burdens—suggesting Maranhão remained significantly above the national average (23, 24).

Balsas, the largest city in southern Maranhão, serves as the health referral center for 13 surrounding municipalities, covering a population of approximately 250,000 (25, 26).

This implementation project in Balsas was conducted as part of a larger multi-site study designed to assess and strengthen family planning services in Zika-affected areas, the protocol for which has been published previously (27). The present manuscript reports specifically on the service strengthening and task-sharing intervention implemented in Balsas, Maranhão, and does not include facility assessment or follow-up evaluation data from other sites described in the broader protocol.

The intervention followed a phased, four-step implementation process, grounded in the Exploration, Preparation, Implementation, Sustainment (EPIS) framework (28). This structured approach allowed for adaptation to the local context, addressing health system challenges and enhancing service delivery through a mix of capacity building, stakeholder engagement, and evidence-based decision-making (29, 30).

The Ministry of Health led the project with technical support from WHO and contributions from local stakeholders. The core implementation team included:

- A local health administrator from Balsas Health Department, overseeing human resources and supply logistics.
- The City Coordinator for Primary Health Care, facilitating coordination across facilities.
- A nurse managing day-to-day operations, family planning counseling, patient flow, and data collection at the lead facility.
- A gynecology and obstetrics consultant, providing clinical training and supervision, particularly for IUD insertion and removal, aligned with WHO guidelines.

The Women's Health Program, based at the Dr. Rosy Kury Municipal Hospital in Balsas, was designated as the lead facility and developed into a center of excellence for family planning training and service delivery. This hospital serves as the health referral center for Balsas and 13 surrounding municipalities.

## 3 Four-phase implementation approach using the EPIS framework

**Figure 1:**
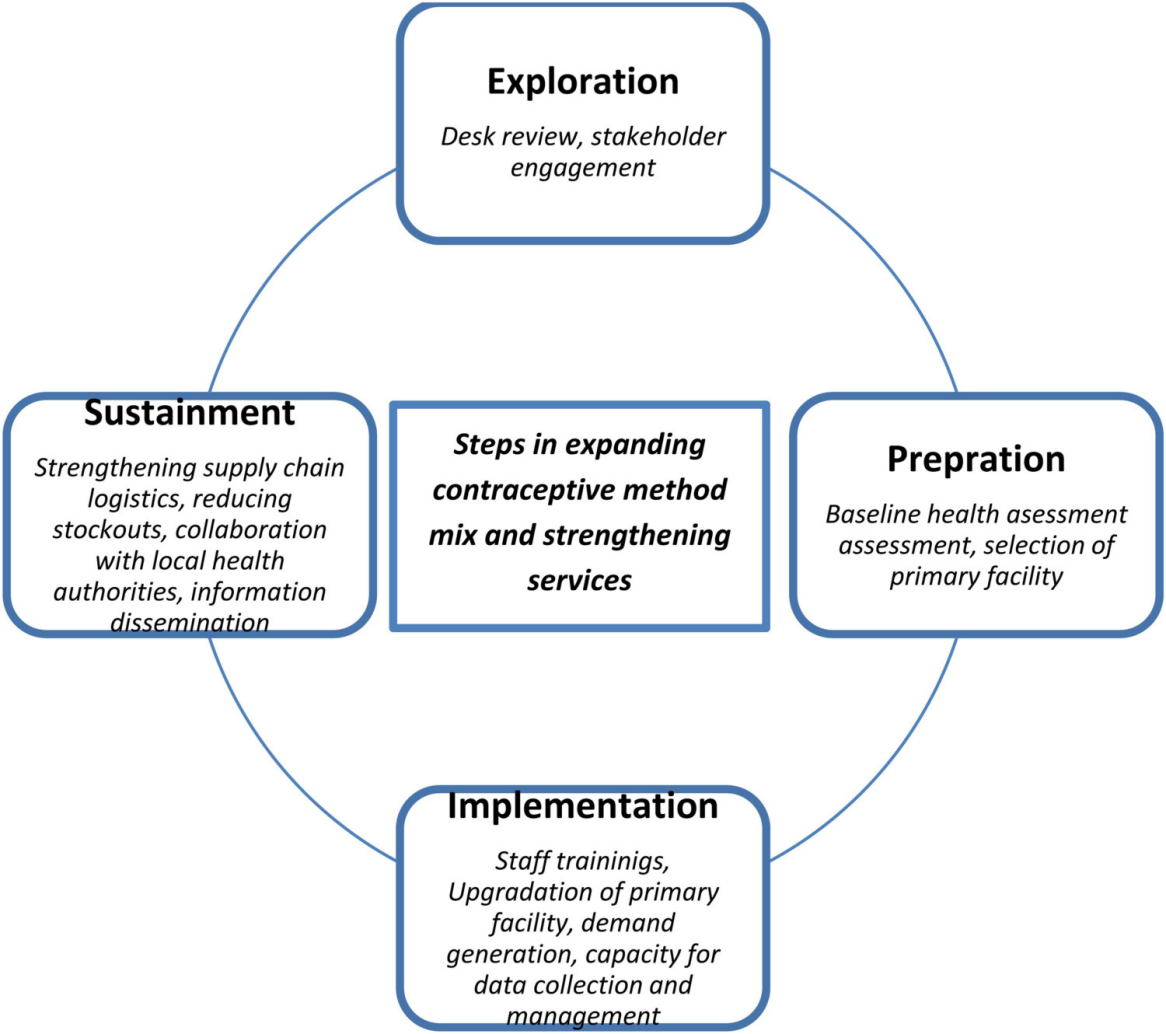
The four steps implementation approach.

To guide the rollout of strengthened family planning services in Balsas, the project adopted a structured implementation approach informed by the EPIS (Exploration, Preparation, Implementation, Sustainment) framework. This phased strategy enabled systematic planning, context-specific adaptation, and progressive scale-up of services (see Figure 1).

### 3.1 Exploration

The exploration phase comprised a desk review and structured stakeholder engagement to identify barriers and inform intervention design.

#### 3.1.1 Desk review findings

A comprehensive review of national policies and municipal health data revealed that: (1) while WHO medical eligibility criteria for contraceptive use had been officially adopted by the Ministry of Health, no structured training program existed for non-specialist providers in IUD insertion/removal; (2) municipal health data (2015–2016) showed that 73% of family planning consultations in Balsas resulted in provision of short-acting methods (oral contraceptives or injectables), despite documented user dissatisfaction and high discontinuation rates; and (3) referral pattern analysis indicated that women seeking IUDs traveled an average of 47 km and waited 4–6 months for appointments with the sole municipal gynecologist.

#### 3.1.2 Stakeholder engagement

Between March and May 2017, the project team conducted three structured meetings with the Municipal Health Secretary and technical team, two workshops with primary care facility coordinators from all 13 municipalities in the health region, and individual interviews with 12 community health workers serving urban and rural catchments. Key feedback that guided intervention contextualization included:

- “Women ask about IUDs but we cannot offer them here. They don't return when referred to the hospital.” (Decision to locate IUD services at the primary care level rather than centralizing at the hospital).
- “Nurses already do cervical cancer screening—they have the skills for IUD insertion.” (Inclusion of nurses as IUD providers, not only counselors).
- “We cannot close clinics for training; staff must be trained without disrupting services.” (Adoption of a cascade training model with on-site mentoring rather than off-site courses).
- “Supplies arrive but no one is trained to use them.” (Procurement linked directly to competency certification).
- “Adolescents don't come because they think family planning is for married women.” (Establishment of dedicated adolescent hours and development of youth-friendly counseling materials).

#### 3.1.3 Local ownership mechanisms

The Municipal Health Department co-chaired the project steering committee, local providers reviewed and approved all training materials, and the Balsas Health Department contributed office space and dedicated 0.5 FTE nurse coordinator.

### 3.2 Preparation

A baseline health system assessment was conducted from June to July 2017 across all 26 primary care units in Balsas municipality using an adapted WHO Service Availability and Readiness Assessment (SARA) tool. Additional methods included a self-administered provider survey (47 physicians and nurses, 82% response rate), four focus group discussions (FGDs) with women of reproductive age stratified by age (18–24, 25–35, 36–49) and urban/rural residence, two FGDs with adolescent women (15–17) recruited through schools (total 58 participants), and 15 key informant interviews with community health workers and 8 with pharmacists.

#### 3.2.1 Key findings

##### 3.2.1.1 Facility-level barriers

- Facilities with IUD insertion equipment: 0 of 26 (0%).
- Facilities with IUDs in stock (past 12 months): 0 of 26 (0%).
- Facilities with a trained IUD insertion provider: 1 of 26 (3.8%)—the sole hospital gynecologist.
- Facilities with contraceptive counseling protocols available: 3 of 26 (11.5%).
- Facilities with adolescent-specific services: 2 of 26 (7.7%).

##### 3.2.1.2 Provider-level barriers

- 94% of nurses and 78% of physicians incorrectly believed IUD insertion required gynecologist certification.
- 68% of providers reported never receiving training on any LARC method.
- 41% of physicians expressed concern that IUD insertion exceeded their scope of practice (pre-training).

##### 3.2.1.3 Community-identified barriers (FGDs)

*Thematic analysis revealed four principal constraints*:

- *Access constraints*: “I went to the clinic three times. First, they said the doctor wasn't there. Second, they said the IUD wasn't available. Third, they gave me pills and said come back next year.” (FGD participant, age 29, urban)
- *Information gaps and myths*: Many FGD participants had heard IUDs “cause infection” or “lead to infertility”; also believed IUDs were only for women who had already completed childbearing. Adolescent participants uniformly reported no school-based contraceptive education addressing LARCs.
- *Provider attitudes*: “The doctor told me IUD is for older women, that I'm too young. He said I should use pills first.” (FGD participant, age 19, urban)
- *Rural–urban disparity*: “To get an IUD, I must travel to Balsas city. The bus leaves at 4 a.m. I lose a day of work and pay 40 reais. Then they say come back next month.” (FGD participant, age 34, rural).

#### 3.2.2 Catalytic funding and sustainability design

Based on these findings, the Women's Health Program, located at the Dr. Rosy Kury Municipal Hospital, was selected as the initial site for service strengthening. One-time catalytic funding of USD 55,700 (2017–2018) supported:

- *Training and mentoring (USD 24,500)*: Facilitator fees, travel for on-site supervision; used to certify local trainers and embed cascade training in municipal continuing education.
- *Equipment and supplies (USD 18,200)*: 10 complete IUD insertion kits, pelvic models, procedure tables; all equipment retained as municipal assets.
- *Data systems (USD 7,800)*: 3 computers, electronic registry software adaptation; systems integrated with the municipal Primary Care Information System; maintenance budgeted by municipality.
- *Demand generation (USD 5,200)*: Printed materials, waiting room displays; materials adapted for local reproduction; community health worker integration.

Sustainability was designed through: (1) cascade training model creating six local trainers; (2) equipment retention as municipal assets; (3) integration of IUD consumables into municipal procurement budgets (the municipality now budgets annually for 800 IUD kits); and (4) incorporation of IUD services into the Municipal Health Plan (2020–2023).

### 3.3 Implementation

#### 3.3.1 Core team training

In August 2017, a core team of 10 providers from the Women's Health Program completed a 40-hour competency-based training covering WHO medical eligibility criteria (5th edition), counseling protocols for LARC methods, infection prevention, instrument handling, and IUD insertion/removal with pelvic model practicum (minimum 10 supervised insertions). The core team comprised 3 general practitioners (GPs), 4 nurses, and 3 nurse assistants. Post-training, GPs and nurses assumed responsibility for IUD insertion/removal; nurse assistants focused on counseling, patient flow, and instrument sterilization.

#### 3.3.2 Monitoring and data use training

To enable service monitoring, 4 core team nurses, 2 administrative staff from the Municipal Health Department, and 1 primary care coordinator received training on contraceptive uptake monitoring and analysis. Rather than creating a parallel reporting system, the project modified existing instruments by adding IUD-specific fields to the municipality's standardized family planning encounter form and mapped variables to Brazil's Primary Care Information System (SISAB). A simple Excel-based dashboard was developed and updated monthly, displaying IUD insertions by facility and provider type, method mix, client age distribution (including adolescents), and wait times. The dashboard was presented at monthly municipal health meetings, enabling facility managers to compare performance and stimulating healthy competition. Examples of data use: (1) when October 2017 data showed 78% of IUD insertions occurred at the hospital while rural clinics had near-zero insertions, cascade training was accelerated to three rural facilities and a mobile insertion team deployed; (2) adolescent IUD uptake <5% led to establishment of adolescent-dedicated hours and revised community health worker messaging; (3) 23% no-show rate for 30-day follow-up prompted revision of follow-up protocol (phone contact at 7 days, home visit by CHW if unreachable); (4) projected IUD stock depletion triggered emergency procurement.

#### 3.3.3 Sixty-day follow-up training (October 2017)

A skills consolidation and problem-solving workshop was held with all 10 core team members plus 3 additional nurses identified as potential cascade trainers. Challenges addressed included:

- Longer insertion times than expected: The team extended supervised practicum, peer mentoring pairs.
- High no-show rate for scheduled insertions: The team centralized scheduling by dedicated nurse assistant.
- Sterilization instrument delays due to shared autoclave: The team dedicated IUD instrument set, scheduled sterilization during low-volume hours.
- Provider anxiety about perforation risk: Minimum competency raised to 15 supervised insertions; on-call gynecologist backup protocol.
- Adolescent reluctance to attend regular clinic hours: Dedicated adolescent hours (Tuesdays 4–7 p.m.), youth peer educators involved.

#### 3.3.4 Cascade training reach

Between September 2017 and December 2018, cascade training expanded IUD services to all 26 primary care facilities in Balsas and four neighboring municipalities. Training levels and reach:

- *Level 1 (core team)*: 1 facility, 10 providers (3 GPs, 4 nurses, 3 assistants).
- *Level 2 (Balsas urban facilities)*: 23 facilities, 46 providers (14 GPs, 21 nurses, 11 assistants).
- *Level 3 (Balsas rural facilities)*: 3 facilities, 8 providers (3 GPs, 5 nurses).
- *Level 4 (neighboring municipalities)*: 4 municipalities, 16 providers (7 GPs, 9 nurses). *Total*: 80 providers trained across 26 facilities and 4 municipalities. Training duration varied: core team 40 h + 20 h supervised clinical practice; urban facilities 24 h (didactic + pelvic model); rural facilities 16 h (condensed didactic, extended hands-on); neighboring municipalities 24 h (Balsas-based).

#### 3.3.5 Provider turnover and mitigation

During the intervention period (2017–2018), turnover rates were 23.5% (4/17) among trained GPs, 19.2% (5/26) among trained nurses, and 27.3% (3/11) among nurse assistants. Mitigation strategies included: (1) redundancy building—minimum 2 trained providers per facility; (2) local trainer cadre- 6 providers (2 GPs, 4 nurses) certified as trainers, reducing dependence on external consultants; (3) rapid onboarding protocol-10-day competency-based training for replacement staff with supervised insertions; (4) contract incentives—municipal health authorities prioritized retention of trained providers through temporary contracts. As a result, service continuity was maintained; for example, when a rural GP left, a trained nurse stepped in and a new GP was trained within 45 days.

### 3.4 Sustainment

#### 3.4.1 Supply chain strengthening

A baseline supply chain assessment (August 2017) identified critical gaps: no dedicated IUD procurement; stock not visible at facility level (central warehouse had IUDs but facilities reported “out of stock” due to lack of trained providers and ordering protocols); no consumption data recorded; and irregular distribution. Interventions implemented:

- *Procurement reform*: IUDs included in municipal annual pharmaceutical procurement plan for the first time (2018), with forecast based on service target (800 IUDs/year) and dedicated budget line (R$ 48,000 annually, approx. USD 9,600).
- *Inventory management*: Minimum stock level (2-month supply, 130 IUDs) established at central warehouse; reorder trigger at 1-month supply; monthly stock visibility dashboard distributed to all facilities.
- *Facility-level supply assurance*: Each trained facility received initial IUD kit (10 devices) upon provider certification; consumption-based resupply using monthly consumption reports; rural clinics maintain 1-month buffer.
- *Distribution logistics*: IUDs included in routine pharmaceutical delivery schedule (biweekly); emergency transport available within 24 h (used twice in 2018).
- *Accountability measures*: Signed receipt required for all IUD transfers; quarterly inventory reconciliation.

This all resulted in zero stockouts of copper IUDs at trained facilities from November 2018 through December 2019; 94% of facilities maintained minimum stock levels (vs. 0% at baseline); expiration losses reduced to zero (2018–2019).

#### 3.4.2 Supportive supervision

A deliberate model of external supportive supervision was adopted, conducted by municipal health authorities rather than facility staff, to ensure objectivity, cross-facility learning, systemic problem identification, and accountability. The existing Municipal Health Department supervision system was enhanced: The supervision protocol included a 12-item IUD service quality checklist, with a minimum of two counseling sessions observed per visit. Monthly verification of IUD insertion logs against service records was conducted, along with a brief knowledge assessment that included remediation referrals. Standardized supervision forms were completed, with a copy left at the facility, and a tracking log at the municipal level monitored follow-up on action items. Post-project, supervision frequency was tiered: quarterly for facilities with more than 20 IUD insertions per month, semi-annually for lower-volume facilities, and annually for remote rural clinics.

#### 3.4.3 Gaps identified and course corrections

Throughout implementation, few gaps were identified and addressed through data-driven corrections. Low IUD uptake among adolescents in October 2017 prompted dedicated hours, peer educators, and school outreach. The following month, rural providers lacking confidence received mobile mentoring with supervised insertions. Inadequate counseling time in December led to group sessions paired with individual consultations. When IUD strings were not visualized at follow-up in January 2018, protocols and job aids were reinforced. High 30-day loss to follow-up in February triggered active tracking via phone contact and community health worker visits. Ongoing provider turnover was managed through redundancy planning and rapid replacement. Stock not reaching facilities in March was resolved with biweekly distributions. Underutilized adolescent hours in April were extended and promoted on social media. Finally, GP reluctance to maintain skills in June was addressed by requiring five monthly insertions and pairing low-volume GPs with high-volume nurses.

## 4 Results

The implementation of the four-step strategy led to meaningful changes in the structure, delivery, and accessibility of family planning services in Balsas, despite the absence of routine data on contraceptive prevalence or expected demand at the municipal level. The intervention focused on building health system capacity, expanding method availability, and increasing access to quality contraceptive counseling and services through a structured, phased approach.

As a result of the project, the facility reorganized its family planning service model to emphasize informed method choice, establishing team-based care that included gynecologists, general practitioners, nurses, and nurse assistants. Nurses assumed a central role in providing comprehensive contraceptive counseling and immediate method provision, which significantly improved the efficiency and accessibility of services. Previously, clients were required to attend separate appointments at different facilities to obtain their chosen method. Service records from the Dr. Rosy Kury Municipal Hospital showed that the number of voluntary IUD insertions were only eight from 2013 to 2017. By December 2018, following training and service reorganization, the same facility reported approximately 1,468 voluntary IUD insertions, marking a dramatic increase in the availability and uptake of long-acting reversible contraception.

The figure of 1,468 IUD insertions in 2018 was triangulated from three data sources: hospital surgical logbook (1,412 insertions), pharmacy dispensing records (1,500 IUDs dispensed), and the family planning clinic register (1,468 insertions reconciled). The dramatic increase reflects the near-complete absence of IUD services at baseline (8 insertions in 5 years) rather than exceptional demand, and is consistent with the removal of severe supply-side restrictions. All insertions followed standardized counseling on contraceptive method options, and clients voluntarily selected the IUD; no quotas or method-specific targets were used.

A key component of the intervention was capacity building and task sharing. One of the most significant changes involved transferring the responsibility for IUD insertions from specialists to general practitioners (GPs). By the end of the intervention, the single gynecologist who had previously performed IUD insertions had been replaced by two trained GPs who independently managed these services. In total, ten GPs across the municipal health system, including those in rural clinics up to 210 km from Balsas city, had been trained and were routinely performing IUD insertions. This redistribution of tasks enhanced service coverage and sustainability while maintaining quality standards through structured training and supportive supervision.

The project's expansion phase extended its impact further through a cascade training model. Health professionals including nurses and GPs from all 26 primary care facilities in Balsas, including three rural satellite units, received training from the implementation team at the primary center. In the past, no IUD insertions were offered in these health facilities due to lack of training and instruments. The training covered the WHO medical eligibility criteria, contraceptive method efficacy, safety and side effects, counseling skills, and hands-on instruction in IUD insertion and removal. Four neighboring municipalities that had previously lacked IUD services also began offering comprehensive family planning, including IUD insertions, for the first time. In total, 80 providers (GPs, nurses, and nurse assistants) were trained through the cascade model, including providers from all 26 Balsas facilities and four neighboring municipalities. Despite turnover, service continuity was maintained through redundancy and rapid onboarding. Although precise utilization data were not systematically collected, local health authorities reported a noticeable increase in contraceptive uptake following the expansion.

Logistics and quality monitoring were also strengthened throughout the intervention. Supply chain strengthening resulted in zero stockouts of copper IUDs at trained facilities from November 2018 through December 2019, compared to 0% of facilities with IUDs in stock at baseline. Expiration losses were eliminated, and majority of facilities maintained minimum stock levels by endline. Additionally, facility-level data collection practices improved, enabling more effective monitoring and supporting data-informed decision-making. Despite these advances, broader population-level indicators such as contraceptive prevalence and unmet need for family planning remain unavailable for the municipality.

## 5 Discussion

This implementation experience in Balsas, Maranhão, illustrates how targeted, context-specific interventions can effectively strengthen family planning services in resource-limited settings (29, 30). By applying a structured implementation science approach, the project addressed long-standing systemic barriers to contraceptive access particularly for long-acting reversible contraceptives (LARCs) within Brazil's public health system (SUS) (27).

A key achievement was the shift from a fragmented, specialist-dependent service model to a more inclusive and decentralized approach. Empowering nurses and general practitioners to counsel and provide a broader range of methods, especially copper IUDs, greatly expanded service coverage. This shift not only improved efficiency and access but also ensured that contraceptive care became more client-centered, with a stronger emphasis on informed choice and method availability at the point of care.

Importantly, this intervention was conducted in a region with a historically weak health infrastructure, high adolescent fertility, and persistent social inequalities. The dramatic increase in voluntary IUD insertions, from eight insertions over past 3 years to approximately 1.468 in just over 1 year demonstrates the effectiveness of the model. The term “voluntary” is used to distinguish these insertions from non-consenting procedures and to indicate that all clients received standardized counseling on method options, efficacy, side effects, and duration before selecting the IUD. No quotas, incentives, or method-specific targets were employed. However, the manuscript does not present data on the proportion of counseled clients who chose IUD vs. other methods, client satisfaction, or method continuation; direct observation of counseling was conducted but not systematically measured. Moreover, the successful task-sharing to general practitioners, including those in rural areas, suggests strong potential for replication and scalability across similar contexts in Brazil and the wider Latin American region (31).

The project also underscores the importance of ongoing capacity building and supportive supervision. The cascade training model allowed for rapid diffusion of skills and knowledge, reaching all 26 primary care facilities in Balsas and extending impact into neighboring municipalities. Collaboration with local health authorities throughout the process fostered ownership, accountability, and sustainability.

The rapid increase in IUD uptake, from near-zero to 1,468 insertions in 12 months, demonstrates that when supply-side barriers are removed, substantial latent demand exists even in settings with widespread contraceptive misinformation. However, the initiative also highlights ongoing challenges. Despite improved service delivery and facility-level monitoring, the lack of routine data on contraceptive prevalence and unmet need at the municipal level limits the ability to fully assess impact on population-level outcomes. Strengthening health information systems remains a critical next step to track long-term progress and inform policy.

This implementation effort needs to be interpreted carefully as it was conducted in a single geographic area, limiting the generalizability of findings to other regions with different health system structures or population needs. While the intervention addressed key service delivery gaps, structural barriers such as long term supply chain constraints, and sociocultural norms affecting contraceptive use were not fully resolved.

A further limitation is the absence of municipal-level data on uptake of short-acting contraceptive methods during the intervention period. It is therefore not possible to determine whether IUD uptake represented a net increase in contraceptive coverage or substitution from other methods. Nonetheless, given the very low baseline IUD use (1.8% nationally) and high unplanned pregnancy rate, increased IUD access addresses an important method gap regardless of substitution effects.

Additionally, while the intervention improved service readiness and provider confidence, persistent sociocultural barriers such as myths about IUDs and resistance to use among certain subgroups may continue to affect uptake. Demand generation and community engagement will be essential to sustaining gains and addressing these barriers moving forward.

## 6 Conclusion

This study demonstrates that systematic, phased implementation of evidence-based strategies rooted in local engagement and supported by national leadership can significantly enhance family planning service delivery in low-resource settings. The Balsas experience offers a replicable model for expanding contraceptive access, especially for LARCs, in underserved areas. Continued investment in provider training, health system readiness, and data systems will be vital to sustain improvements and move toward equitable reproductive health for all.

## Data Availability

The raw data supporting the conclusions of this article will be made available by the authors, without undue reservation.

## References

[1] Qiao L Martelli CMT Raja AI Sanchez Clemente N de Araùjo TVB Ximenes RAA Epidemic preparedness: prenatal Zika virus screening during the next epidemic. BMJ Glob Health. (2021) 6(6):e005332. 10.1136/bmjgh-2021-005332 34117012 PMC8202108

[2] Petersen LR Jamieson DJ Powers AM Honein MA. Zika virus. N Engl J Med. (2016) 374(16):1552–63. 10.1056/NEJMra1602113 27028561

[3] Hennessey M Fischer M Staples JE. Zika virus spreads to new areas—region of the Americas, May 2015–January 2016. Morb Mortal Wkly Rep. (2016) 65(3):55–8. 10.15585/mmwr.mm6503e1 26820163

[4] UN DESA. World contraceptive use 2022. United Nations, Department of Economic and Social Affai (2022).

[5] DHS Program. Demographic and health surveys: Brazil (2020).

[6] UNFPA. World Population Dashboard Brazil. (2019). Available online at: https://www.unfpa.org/data/world-population/BR (Accessed May 9, 2025).

[7] Theme-Filha MM Baldisserotto ML Fraga ACSA Ayers S da Gama SGN Leal MC. Factors as-sociated with unintended pregnancy in Brazil: cross-sectional results from the birth in Brazil national survey, 2011/2012. Reprod Health. (2016) 13(Suppl 3):118. 10.1186/s12978-016-0227-8 27766945 PMC5073899

[8] Pesquisa Nacional de Demografia e Saúde da Criança e da Mulher. Banco de dados [National Demographic and Health of Children and Women: Database] [webpage on the Internet]. Ministry of Health of Brazil; 2008. Available online at: http://bvsms.saude.gov.br/bvs/pnds/banco_dados.php (Accessed May 9, 2025).

[9] Sedgh G Singh S Hussain R. Intended and unintended pregnancies worldwide in 2012 and recent trends. Stud Fam Plann. (2014) 45(3):301–14. 10.1111/j.1728-4465.2014.00393.x 25207494 PMC4727534

[10] Borges ALV Dos Santos OA Fujimori E. Concordance between intention to use and current use of contraceptives among six–month postpartum women in Brazil: the role of unplanned pregnancy. Midwifery. (2018) 56:94–101. 10.1016/j.midw.2017.10.015 29096285

[11] Rocha RC de Souza E Soares EP Nogueira ES Chambô Filho A Guazzelli CA. Prematurity and low birth weight among Brazilian adolescents and young adults. J Pediatr Adolesc Gynecol. (2010) 23(3):142–5. 10.1016/j.jpag.2009.08.011 19822446

[12] Paim J Travassos C Almeida C Bahia L Macinko J. The Brazilian health system: history, advances, and challenges. Lancet. (2011) 377(9779):1778–97. 10.1016/S0140-6736(11)60054-8 21561655

[13] Massuda A Hone T Leles FAG de Castro MC Atun R. The Brazilian health system at crossroads: progress, crisis, and resilience. BMJ Glob Health. (2018) 3(4):e000829. 10.1136/bmjgh-2018-000829 29997906 PMC6035510

[14] Andrade MV Noronha KVMS Menezes RM Souza MN Reis CB Martins DR Desigualdade socioeconômica no acesso aos serviços de saú-de no Brasil: um estudo comparativo entre as regiões brasileiras em 1998 e 2008. Econ Apl. (2013) 17:623–45. 10.1590/S1413-80502013000400005

[15] Costa SH Pillai VK Noronha K de Oliveira LG. Inequities in contraceptive access in Brazil. Rev Saude Publica. (2021) 55:86.34852166

[16] Ministério da Saúde. Cadernos de Atenção Básica—Saúde Sexual e Reprodutiva (2020).

[17] Machado AKF Gräf DD Höfs F Hellwig F Barros KS Moreira LR Prevalence and inequalities in contraceptive use among adolescents and young women: data from a birth cohort in Brazil. Cad Saude Publica. (2021) 37(10):e00335720. 10.1590/0102-311X00335720 34787284

[18] Diniz D Costa T Madeiro A. Female sterilization and inequality in Brazil. Reprod Health Matters. (2017) 25(50):132–40.

[19] Bahamondes L Fernandes A Monteiro I. Barriers to implementing and consolidating a family plan-ning program that would meet Brazilian needs. Rev Bras Ginecol Obstet. (2017) 39(8):373–5. 10.1055/s-0037-1604423 28772333 PMC10309472

[20] Bahamondes L Bottura BF Bahamondes MV Goncalves MP Correia VM Espejo-Arce X Estimated disability-adjusted life years averted by long-term provision of long-acting contraceptive methods in a Brazilian clinic. Hum Reprod. (2014) 29(10):2163–70. 10.1093/humrep/deu191 25085802

[21] Harris LH Silverman NS Marshall MF. The paradigm of the paradox: women, pregnant women, and the unequal burdens of the Zika virus pandemic. Am J Bioeth. (2016) 16(5):1–4. 10.1080/15265161.2016.1177367 27111356

[22] Schuck-Paim C Lopez D Simonsen L Alonso W. Unintended pregnancies in Brazil—a challenge for the recommendation to delay pregnancy due to Zika. PLoS Curr. (2016) 8. 10.1371/currents.outbreaks.7038a6813f734c1db547240c2a0ba291 28515967 PMC4866532

[23] Szwarcwald CL Escalante JJC Rabello Neto DL Souza Junior PRB Victora CG. Methodological issues. Cad Saúde Pública. (2014) 30(Suppl 1):S71–83. 10.1590/0102-311X00125313 25167192

[24] Oliveira IVG Maranhão TA Sousa GJB Silva TL Rocha MIF Frota MMC Maternal mortality in northeast Brazil 2009–2019: spatial distribution, trend and associated factors. Epidemiol Serv Saúde. (2023) 32(3):30. 10.1590/S2237-96222023000300009.EN PMC10615180 37909520

[25] Brasil Maranhão Balsas. Available online at: https://cidades.ibge.gov.br/brasil/ma/balsas/panorama (Accessed May 9, 2025).

[26] Instituto Brasileiro de Geografia e Estatistica. Balsas: Municipio do estado do Maranhao, no Brasil (2018).

[27] Ali M Folz R Miller K Johnson BR Kiarie J. A study protocol for facility assessment and follow-up evaluations of the barriers to access, availability, utilization and readiness of contraception, abortion and postabortion services in Zika affected areas. Reprod Health. (2017) 14(1):18. 10.1186/s12978-017-0283-8 28153043 PMC5288873

[28] Moullin JC Dickson KS Stadnick NA Rabin B Aarons GA. Systematic review of the exploration, preparation, implementation, sustainment (EPIS) framework. Implement Sci. (2019) 14(1):1. 10.1186/s13012-018-0842-6 30611302 PMC6321673

[29] Aarons GA Hurlburt M Horwitz SM. Advancing a conceptual model of evidence-based practice implementation in public service sectors. Adm Policy Ment Hlth. (2011) 38(1):4–23. 10.1007/s10488-010-0327-7 PMC3025110 21197565

[30] Aarons GA Ehrhart MG Farahnak LR Sklar M. Aligning leadership across systems and organizations to develop a strategic climate for evidence based practice implementation. Annu Rev Public Health. (2014) 35(1):255–74. 10.1146/annurev-publhealth-032013-182447 24641560 PMC4348088

[31] Cleland J Ali M Benova L Daniele M. The promotion of intrauterine contraception in low- and middle-income countries: a narrative review. Contraception. (2017) 95(6):519–28. 10.1016/j.contraception.2017.03.009 28365165

